# Social support, family resilience and psychological resilience among maintenance hemodialysis patients: a longitudinal study

**DOI:** 10.1186/s12888-024-05526-4

**Published:** 2024-01-26

**Authors:** Yuxin Wang, Yuan Qiu, Liya Ren, Hao Jiang, Meijia Chen, Chaoqun Dong

**Affiliations:** 1 School of Nursing, Wenzhou Medical University, University Town, Chashan, Wenzhou, Zhejiang 325035 China; 2 Zhejiang Tourism and Health College, Zhoushan, China; 3 The Second School of Medicine, Wenzhou Medical University, Wenzhou, China

**Keywords:** Maintenance hemodialysis, Social support, Family resilience, Psychological resilience, Cross-lagged analysis, Longitudinal study

## Abstract

**Background:**

Psychological distress is common in maintenance hemodialysis patients, and high psychological resilience can promote psychological well-being. The current research focuses on psychological resilience protective factors such as family resilience and social support. However, the trajectories of psychological resilience, family resilience, and social support over time and their longitudinal relationships in maintenance hemodialysis patients have not been fully explored yet. Therefore, this study aims to explore the longitudinal relationship between these factors.

**Methods:**

Patients who received regular hemodialysis treatment for more than three months at dialysis centers of three tertiary hospitals in Zhejiang, China, were recruited from September to December 2020. A total of 252 patients who met the inclusion and exclusion criteria completed three follow-up surveys, including social support, family resilience, and psychological resilience assessments. A repeated measures ANOVA was used to explore differences in their respective scores at different time points. The cross-lagged analysis was performed in AMOS using the maximum likelihood method to examine the the reciprocal predictive relationships between these factors.

**Results:**

Social support and psychological resilience remained relatively stable over time, whereas family resilience indicated a little increasing trend. According to the cross-lagged analysis, higher T1 social support predicted higher family resilience at T2 [β = 0.123, 95% CI (0.026–0.244)]. Further, the effects of T2 social support to T3 family resilience [β = 0.194, 95%CI (0.039–0.335)] and psychological resilience [β = 0.205, 95%CI (0.049–0.354)] were significant. Finally, the effects of T2 family resilience to T3 social support [β = 0.122, 95%CI (0.010–0.225)] and psychological resilience [β = 0.244, 95%CI (0.119–0.359)] were also significant.

**Conclusions:**

The study showed that the directionality of the relationship appears to be from social support or family resilience to patients’ psychological resilience but not vice versa. This finding reminds healthcare professionals to emphasize the vital role of social and family resources in providing appropriate support and interventions for maintenance hemodialysis patients to promote psychological resilience and mental health development.

**Supplementary Information:**

The online version contains supplementary material available at 10.1186/s12888-024-05526-4.

## Background

The prevalence of end-stage renal disease (ESRD) in China has been on the rise since 2013 [1], with complicated symptoms and high mortality rates [2], seriously threatening the lives of patients. Maintenance hemodialysis (MHD), which is defined as receiving hemodialysis for more than three months [3], is the primary type of dialysis for ESRD [4]. For most patients with ESRD, MHD provides hope for sustaining life. According to statistics, 553,000 patients with MHD were in China as of 2019 [4]. The number of dialysis patients in China is predicted to increase further through 2025 [1], bringing a significant financial and ecological burden to the healthcare system [5]. MHD patients typically undergo more than two dialysis sessions per week [6]. Due to repeated punctures, fluid and dietary restrictions, and dialysis complications in the process of frequent dialysis treatments, it is highly likely for MHD patients to suffer from psychological distress, such as anxiety [7], depression [8], and feelings of symptom burden [9]. Moreover, the financial burden brought by weekly dialysis, long-term medication, and laboratory tests imposes significant financial stress on the families, potentially causing guilt and exacerbating the patients’ psychological pressure [10]. The literature pointed to a high incidence of depression in hemodialysis patients compared with the general population, which was significantly correlated with a heightened risk of mortality, hospitalization, and poor adherence and quality of life [11, 12]. Therefore, addressing the psychological problems of MHD patients and promoting their psychological adaptation is particularly necessary.

Psychological resilience, defined as the capacity for recovery and “bouncing back” from adversity or significant sources of stress, is a personal trait that can be developed [13]. Evidence showed that psychological resilience may alleviate depressive symptoms generated by disease stress in hemodialysis patients and promote their psychological well-being [8]. Moreover, psychological resilience helps patients manage stressful situations and improve their quality of life [14]. Kumpfer’s psychological resilience framework states that psychological resilience exhibits dynamic changes influenced by internal and external factors (family, social environment, etc.) [15]. Thus, psychological resilience in MHD patients may change under the influence of disease progression, family characteristics, and social support [16]. A cross-sectional survey in Taiwan found that 83% of MHD patients had a low-to-moderate level of psychological resilience [8], indicating it is urgent to improve the psychological resilience of MHD patients. Consequently, there is a need to explore the protective factors of psychological resilience in patients with MHD in order to promote their psychological status and quality of life.

Ecosystem theory identifies family and social factors as essential influences on an individual’s psychological resilience [17]. Family resilience, which is a crucial measure of family characteristics, is the capacity of families to flexibly utilize internal and external resources to achieve positive adjustment and adaptation in the face of adversity or traumatic events [18]. Several studies have found that family resilience significantly and positively predicts psychological resilience among breast cancer patients [19, 20], undergraduate nursing students [21], and the disabled older population [22]. These studies provide preliminary evidence that family resilience is strongly associated with psychological resilience. Meanwhile, scholars also found that psychological resilience was an important factor influencing family resilience [23]. However, given the cross-sectional design of the studies mentioned above, the directionality of the relationship between psychological resilience and family resilience remains unstudied. The longitudinal transactional models of developmental psychology emphasize the dynamic interactions between the individual and the (family, social) environment [24]. Therefore, this study hypothesizes that psychological resilience and family resilience may positively predict each other.

Social support [25], the combination of emotional and material resources provided by caregivers, medical staff, and other social networks, is believed to be one of the social factors affecting psychological resilience. Wilks and Croom [26] found that social support from family and friends is a protective factor for psychological resilience. High levels of social support promote psychological resilience and positive psychological outcomes to maintain individual physical and mental health [27]. Sippel et al. proposed that positive social support systems can enhance psychological resilience by increasing self-confidence or activating the parasympathetic nervous system and other neurobiological mechanisms [28]. In addition, Wang et al. [29] and Chen et al. [30] found a positive predictive effect of psychological resilience on social support in older caregivers and advanced cancer survivors, respectively. Therefore, this study hypothesized that psychological resilience and social support may positively predict each other.

Ecological systems theory posits that individual development is influenced by interactions across multiple system levels [17], implying that family resilience and social support can interact and positively impact the individual’s psychological resilience. The Walsh family resilience theory identifies social support as a favorable external resource that enhances family resilience [31]. Social support was found to be helpful for families to cope with lung cancer adversity, thereby promoting the development of family resilience [32]. Chen et al. [33] found that family resilience contributed to the perceived social support, which in turn then increased psychological resilience. To summarize, this study hypothesized that family resilience and social support may positively predict each other.

However, the aforementioned studies only looked at the two-by-two association between social support, family resilience, and psychological resilience, and few studies have examined how these three variables interact with each other. Given the difference in sociodemographic and disease characteristics between MHD patients and other patients, previous studies’ findings may not be generalizable to MHD patients. Moreover, due to the nature of cross-sectional studies, prior studies were unable to reveal the directionality of the relationships between these three variables. A cross-lagged model, widely used to analyze how variables interact with each other [34, 35], can use longitudinal data to reveal mutual predictive or quasi-causal associations between two or more variables [36]. Therefore, the present study intends to employ the cross-lagged analysis to explore the reciprocal predictive relationships among social support, family resilience, and psychological resilience in a longitudinal follow-up study among MHD patients, with the purpose of providing scientific evidence to assist healthcare professionals in coordinating family and social factors to develop effective measures to improve the mental health of MHD patients.

## Methods

### Study design and setting

This multi-center longitudinal study was conducted between September 2020 and July 2021 in hemodialysis centers in three comprehensive hospitals in Zhejiang Province, China.

### Participants

**Fig. 1:**
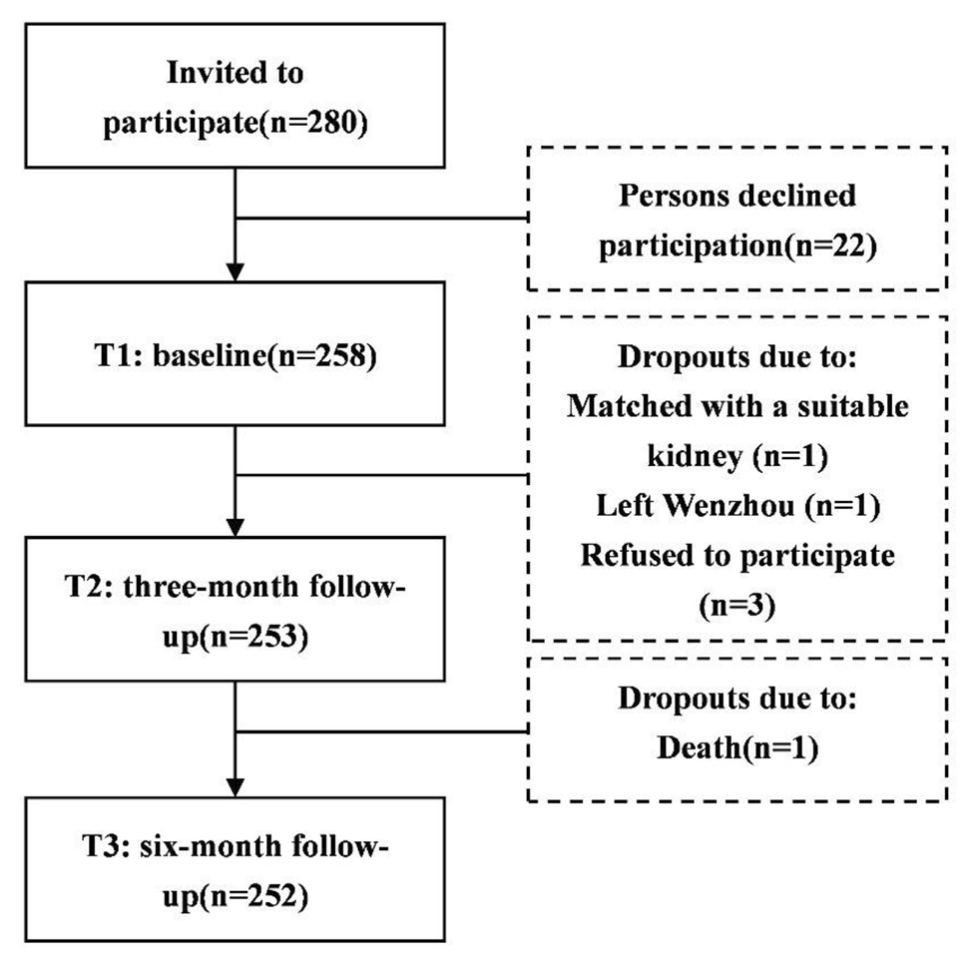
Flowchart of the participants in the study

In this study, MHD patients were recruited by convenience sampling. The eligibility criteria for participants were: (a) aged>18 years, (b) maintaining a regular hemodialysis regimen for at least three months, (c) the treatment frequency at least two times per week, (d) having reading and writing ability, or able to communicate with investigators without difficulty. Patients were excluded if they were diagnosed with psychiatric illnesses or cognitive impairments by clinicians. Boomsma [37] suggested a sample size of no less than 200 when constructing the structural equation model using the maximum likelihood method. And taking a 20% loss rate, the required sample size was expected to be 220. Twenty-two of the 280 patients who met the inclusion criteria declined to participate due to dialysis fatigue or lack of interest. A total of 258 patients were included in the baseline assessment, and 252 patients completed the whole study. Figure 1 illustrates the sampling procedure and dropouts. The effective response rate was 90.4% and 90% at T2 and T3, respectively.

### Procedure

During the baseline assessment (T1), patients who met the inclusion criteria were invited to complete a self-reported questionnaire and were given uniform instructions by a trained researcher. Before administering the survey, the purpose and procedures of the study were presented to patients. After informed consent was obtained, participants were invited to complete the questionnaire in a quiet room. For those unable to complete the questionnaire independently, the researcher provided assistance by reading the items and recording the answers objectively. The entire survey took approximately 20 to 30 min, and the researcher reviewed the questionnaire immediately after completion and collected it on the spot after asking the participants to add any missing items. The respondents were requested to refill the same questionnaire three months (T2) and six months (T3) after the baseline survey (T1). Because maintenance hemodialysis patients were required to have regular hemodialysis in the hospital, the study used the same approach to collect follow-up data in the hospital. Only patients who completed all three waves of the survey were included in the data analysis. The study adheres to the Strengthening the Reporting of Observational Studies in Epidemiology (STROBE) guideline for observational research (Additional file 1).

### Measurements

#### Social support

The patients’ social support in this study was assessed using the Chinese version of the Medical Outcomes Study-Social Support Survey (MOS-SSS) [38]. It consists of 19 items divided into four components: informational/emotional support, tangible support, positive social interactive support, and affectionate support. The overall score ranges from 19 to 95, with higher scores indicating higher levels of social support. The content validity index of the Chinese version of the MOS-SSS was assessed by the expert panel to be 0.82, and the internal consistency Cronbach’s alpha was 0.98 [38]. In this research, Cronbach’s alpha is 0.93–0.94 for the total scale.

#### Family resilience

Family resilience was assessed by the 44-item measure of the Chinese version of the Family Resilience Assessment Scale(C-FRAS) [39]. A 4-point scale ranging from 1 (“strongly disagree”) to 4 (“strongly agree”) consists of four parts: family communication and problem-solving, utilizing social and economic resources, maintaining a positive outlook, and the ability to make meaning of adversity. The total score of the C-FRAS ranged from 44 to 176, with higher scores indicating higher levels of family resilience. The C-FRAS demonstrated good construct validity and acceptable internal consistency (Cronbach’s alpha = 0.96) [39]. In the current study, Cronbach’s alpha is 0.90–0.98 for the total scale.

#### Psychological resilience

Psychological resilience (a dependent variable) was measured by the Chinese version of the Conner and Davidson resilience scale(CD-RISC) [13], which is comprised of three subscales: measuring tenacity, strength, and optimism. This scale has 25 items rated on a 5-point scale from 0 (“not true at all”) to 4 (“true all the time”), and a total score ranging from 0 to 100. Higher scores indicate higher levels of psychological resilience. The Chinese version of CD-RISC was approved to have good construct validity and internal consistency (Cronbach’s alpha = 0.91) [13]. The Cronbach’s alpha is 0.92–0.94 in the current study.

#### Covariates

Based on previous studies [40, 41], patients’ gender (1 = male, 2 = female), age (1 = up to 60 years, 2 = over 60 years), education level (1 = primary school and below, 2 = middle school, 3 = high school/secondary school, 4 = college or higher), employment status (1 = employed, 2 = unemployed), monthly household income per capita (1 = < 2000 RMB, 2 = 2000–4000 RMB, 3 = 4001–6000 RMB, 4 = > 6000 RMB), duration of disease (1 = < 1 year, 2 = 1 ~ < 5 years, 3 = 5 ~ < 10 years, 4 = ≥ 10 years), and duration of hemodialysis ((1 = < 1 year, 2 = 1 ~ < 5 years, 3 = 5 ~ < 10 years, 4 = ≥ 10 years) were included in the study as potential covariates.

### Data analysis

SPSS version 25.0 (IBM Corp., Armonk, NY, USA) and AMOS 22.0 (IBM Corp., Armonk, NY, USA) were used for data analysis. The statistical description was performed using the percentage, mean, and standard deviation (SD), etc. An independent sample t-test and one-way ANOVA test were used to analyze the relationship between demographic characteristics and social support, family resilience, and psychological resilience. Pearson correlation was used to examine the correlations between social support, family resilience, and psychological resilience. The one-way repeated measures ANOVA was used to evaluate for significant differences in social support, family resilience, and psychological resilience across the three-time points. The data were judged to be approximately normally distributed by normality tests (Q-Q plots and P-P plots), and the cross-lagged analysis was conducted using the maximum likelihood method to examine the relationship between social support, family resilience, and psychological resilience in patients with MHD.

Based on previous studies [34, 36] and the hypotheses of this study, five possible pathway models were proposed (see Additional file 1 for details):

M1 - A baseline model with autoregressive paths and cross-sectional correlations;

M2 - A model with cross-lagged paths from prior social support and psychological resilience to later family resilience that examined social support and psychological resilience as predictors of family resilience;

M3 - A model with cross-lagged paths from prior family resilience to later social support and psychological resilience that examined family resilience as predictors of social support and psychological resilience;

M 4 - A model that examined bidirectional associations between social support and family resilience, or between family resilience and psychological resilience.

M 5 - A full model containing all the paths we hypothesized that examined bidirectional associations between social support, family resilience, and psychological resilience.

The optimal model was determined using a combination of the chi-square difference test [42] and the following fit indices: the chi-square/degree of freedom (*χ*^2^/*df*), the Comparative Fit Index (CFI), the Tucker-Lewis Index (TLI), the Root Mean Square Error of Approximation (RMSEA), and the Standardized Root Mean Square Residual (SRMR). Model fit is good when *χ*^2^/*df* < 3, CFI > 0.95, TLI > 0.9, RMSEA < 0.08, and SRMR < 0.08 [43, 44]. *p*-values < 0.05 (two-tailed) were considered statistically significant for all tests.

### Ethical considerations

This study was approved by the Medical Ethics Committee of the hospital (No. 2,020,198). All research procedures were performed in accordance with the Declaration of Helsinki.

## Results

### Characteristics of participants and differences in social support, family resilience, and psychological resilience

The MHD patients’ ages ranged from 19 to 86 years old (mean = 57.40, SD = 13.56). Among them, over half (67.2%) were male. Most patients were married or cohabitating (88.5%) and unemployed (82.1%), with 38.9% of the participants receiving dialysis for more than five years. Further statistical tests revealed that MHD patients with different employment statuses and education levels had significant differences in social support (*t* = 2.908, *p* = 0.004; *F* = 5.297, *p* = 0.001), family resilience (*t* = 2.108, *p* = 0.036; *F* = 2.787, *p* = 0.045), and psychological resilience (*t* = 3.791, *p* < 0.001; *F* = 11.568, *p* < 0.001) at T1, as shown in Table 1. Therefore, the subsequent cross-lagged model included employment status and education level as covariates to control for potential confounding.

**Table 1:** Patients characteristics and differences in participants’ social support, family resilience, and psychological resilience at T1(*n* = 252) (Mean ± SD)

Variable	N(%)	Socail Support	t/F	***p***	Family resilience	t/F	***p***	Psychological Resilience	t/F	***p***
**Age**										
<60	129(51.2%)	65.49 ± 13.76	t=-0.272	0.786	130.66 ± 11.95	t = 0.106	0.916	60.96 ± 15.52	t = 1.869	0.063
≥60	123(48.8%)	65.94 ± 12.77			130.50 ± 11.29			57.43 ± 14.41		
**Gender**										
Male	169(67.2%)	65.16 ± 13.03	t=-0.940	0.348	130.68 ± 10.09	t = 0.189	0.850	60.22 ± 14.95	t = 1.478	0.141
Female	83(32.9%)	66.83 ± 13.72			130.39 ± 14.28			57.24 ± 15.19		
**Employment status**										
Employed	45(17.9%)	70.84 ± 11.07	t = 2.908	0.004	133.87 ± 10.97	t = 2.108	0.036	66.76 ± 13.99	t = 3.791	0.000
Unemployed	207(82.1%)							57.60 ± 14.82		
**Religious Belief**										
No	122(48.4%)	65.34 ± 14.44	t=-0.433	0.665	129.98 ± 12.17	t=-0.794	0.428	59.31 ± 15.91	t = 0.075	0.941
Yes	130(51.6%)	66.06 ± 12.10						59.17 ± 14.28		
**Education level**										
Primary school and below	68(27%)	61.96 ± 15.22	F = 5.297	0.001	127.72 ± 12.07	F = 2.787	0.045	52.69 ± 13.23	F = 11.568	0.000
Middle school	96(38.1%)	66.01 ± 11.66			129.80 ± 7.60			63.21 ± 13.35		
High school/secondary school	57(22.6%)	65.67 ± 12.04			132.54 ± 12.44			69.23 ± 16.59		
College or higher	31(12.3%)	73.10 ± 12.82			135.68 ± 16.60			59.24 ± 15.06		
**Monthly household income per capita**										
<2,000 RMB	36(14.3%)	60.28 ± 13.53	F = 4.246	0.006	127.83 ± 8.51	F = 2.034	0.110	52.81 ± 13.37	F = 8.745	0.000
2,000–4,000 RMB	91(36.1%)	65.96 ± 12.81			129.70 ± 10.48			58.08 ± 14.64		
4,001–6,000 RMB	74(29.4%)	64.93 ± 13.17			130.93 ± 12.22			57.92 ± 13.92		
>6,000 RMB	51(20.2%)	70.24 ± 12.77			133.59 ± 13.92			67.76 ± 15.37		
**Marital status**										
Single/divorced/widow/separated	29(11.5%)	56.14 ± 16.56	t=-4.217	0.000	128.79 ± 10.80	t=-0.882	0.378	53.76 ± 12.50	t=-2.096	0.037
Married/cohabitating	223(88.5)	66.96 ± 12.28			130.82 ± 11.71			59.95 ± 15.25		
**Medical insurance**										
No	5(2%)	69.80 ± 11.78	t = 0.696	0.487	124.20 ± 7.86	t=-1.243	0.215	49.00 ± 14.09	t=-1.639	0.173
Yes	247(98%)				130.71 ± 11.65			59.45 ± 15.04		
**Duration of disease**										
<1 year	13(5.2%)	62.62 ± 13.46	F = 0.957	0.413	129.92 ± 13.60	F = 0.152	0.928	58.46 ± 16.78	F = 0.607	0.611
1 ~ < 5 years	51(20.2%)	68.25 ± 13.27			130.98 ± 10.27			61.69 ± 14.90		
5 ~ < 10 years	54(21.4%)	64.91 ± 14.24			131.31 ± 12.25			57.98 ± 16.31		
≥10 years	134(53.2%)	65.37 ± 12.84			130.20 ± 11.74			58.89 ± 14.50		
**Duration of hemodialysis**										
<1 year	48(19%)	67.17 ± 12.11	F = 3.062	0.029	130.13 ± 10.78	F = 0.559	0.643	58.48 ± 16.03	F = 2.375	0.071
1 ~ < 5 years	106(42.1%)	67.82 ± 12.85			131.61 ± 10.88			62.03 ± 15.08		
5 ~ < 10 years	64(25.4%)	63.53 ± 13.76			129.33 ± 14.73			56.06 ± 15.07		
≥10 years	34(13.5%)	61.18 ± 13.93			130.35 ± 7.84			57.59 ± 12.43		
**Comorbidities**										
No	75(29.8%)	65.67 ± 13.58	F = 0.030	0.971	131.03 ± 13.34	F = 0.095	0.910	58.47 ± 17.13	F = 0.176	0.839
One	117(46.4%)	65.56 ± 12.22			130.51 ± 10.96			59.79 ± 13.83		
Two or more	60(23.8%)	66.07 ± 14.94			130.17 ± 10.65			59.13 ± 14.83		
**Primary caregivers**										
Spouse	208(82.5%)	66.94 ± 11.91	F = 3.768	0.031	131.09 ± 10.83	F = 5.639	0.004	59.81 ± 14.90	F = 1.465	0.225
Offspring	6(2.4%)	70.00 ± 13.73			126.83 ± 1.94			61.17 ± 16.94		
Parents	23(9.1%)	62.83 ± 15.83			131.78 ± 11.40			58.57 ± 12.99		
Sibling	15(6%)	51.40 ± 18.19			123.27 ± 20.17			51.53 ± 18.70		

### Social support, family resilience, and psychological resilience of MHD patients in three waves

**Fig. 2:**
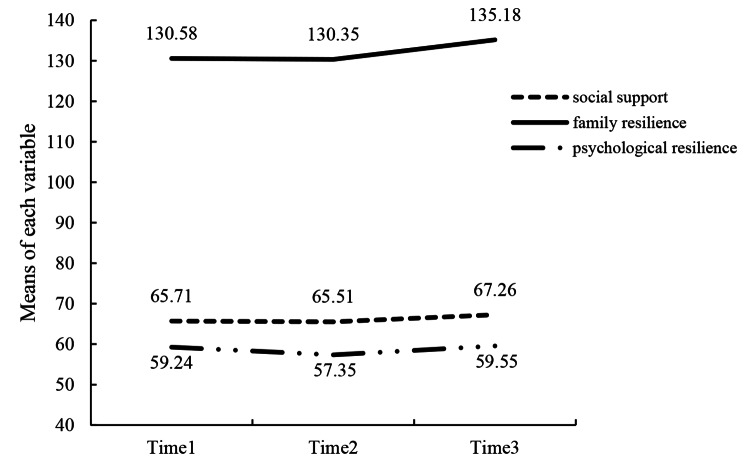
Trends of social support, family resilience, and psychological resilience across the three timepoints

The scores of social support, family resilience, and psychological resilience in the three waves are presented in Table 2. Figure 2 depicts the trends in social support, family resilience, and psychological resilience at the three time points. The social support, family resilience, and psychological resilience at the three time points did not pass the Mauchly’s test of sphericity, *χ*2 (2) = 62.561, 145.823, 13.676, *p* ≤ 0.001, and the Huynh-Feldt correction revealed that there was no statistically significant difference in social support [*F* (1.647, 413.341) = 2.876, *p* = 0.068] and psychological resilience [*F* (1.913, 480.158) = 2.353, *p* = 0.099] between MHD patients at the three time points. However, the level of family resilience changed considerably over the three phases [*F* (1.392, 349.460) = 17.769, *p* < 0.001]. Post hoc analysis with Bonferroni correction revealed that the family resilience increased substantially from T2 to T3 [4.837 (95% CI 2.876–6.798), *p* < 0.001] and T1 to T3 [4.599 (95% CI 2.422–6.777), *p* < 0.001], but not change significantly from T1 to T2 [-0.238 (95% CI 1.311-0835), *p* = 0.662].

**Table 2:** Scores for social support, family resilience, and psychological resilience at the three timepoints (*n* = 252) (Mean ± SD)

Variable	Time 1	Time 2	Time 3
**Social Support**			
Total	65.71 ± 13.26	65.51 ± 12.18	67.26 ± 10.80
Tangible support	16.75 ± 3.40	17.05 ± 3.16	17.5 ± 2.35
Informational/emotional support	24.91 ± 6.07	25.03 ± 5.59	25.54 ± 5.51
Social interactive support	12.76 ± 3.42	12.48 ± 3.21	12.77 ± 3.09
Affectionate support	11.29 ± 2.42	10.95 ± 2.18	11.44 ± 1.93
**Family resilience**			
Total	130.58 ± 11.61	130.35 ± 10.89	135.18 ± 15.10
Family communication and problem solving	81.91 ± 7.45	81.79 ± 7.30	85.81 ± 9.86
Utilizing social and economic resources	21.88 ± 2.51	21.9 ± 2.31	22.19 ± 2.75
Maintaining a positive outlook	17.75 ± 2.07	17.77 ± 2.03	18.03 ± 2.38
Ability to make meaning of adversity	9.04 ± 0.85	8.87 ± 0.88	9.15 ± 1.12
**Psychological Resilience**			
Total	59.24 ± 15.06	57.35 ± 13.72	59.55 ± 14.86
Tenacity	29.78 ± 8.32	28.79 ± 7.39	29.81 ± 8.12
Strength	20.52 ± 5.16	19.63 ± 4.80	20.38 ± 5.08
Optimism	8.94 ± 2.66	8.93 ± 2.49	9.35 ± 2.58

### Relationship between social support, family resilience, and psychological resilience

The intercorrelation of social support, family resilience, and psychological resilience for MHD patients is presented in Table 3. In terms of concurrent correlations, there were significant positive correlations between family resilience, social support, and psychological resilience, both on the T1 measure and on the T2 or T3 measures. In terms of temporal correlations, T1 and T2 social support were correlated with T2 and T3 family resilience, respectively, and vice versa. T1 and T2 social support were correlated with T2 and T3 psychological resilience, respectively, and vice versa. T1 and T2 family resilience were correlated with T2 and T3 psychological resilience, respectively, and vice versa. It shows that there is a concurrent correlation and temporal correlation between the variables, which meets the basic design conditions for cross-lagged analyses.

**Table 3:** Correlations among social support, family resilience, and psychological resilience

Variable	T1 social support	T1 family resilience	T1 psychological resilience	T2 social support	T2 family resilience	T2 psychological resilience	T3 social support	T3 family resilience
T1 social support	1							
T1 family resilience	0.484^**^	1						
T1 psychological resilience	0.486^**^	0.551^**^	1					
T2 social support	0.390^**^	0.195^**^	0.177^**^	1				
T2 family resilience	0.445^**^	0.706^**^	0.433^**^	0.256^**^	1			
T2 psychological resilience	0.278^**^	0.244^**^	0.390^**^	0.520^**^	0.225^**^	1		
T3 social support	0.327^**^	0.135^*^	0.084	0.684^**^	0.284^**^	0.390^**^	1	
T3 family resilience	0.169^**^	0.156^*^	0.064	0.273^**^	0.294^**^	0.181^**^	0.446^**^	1
T3 psychological resilience	0.263^**^	0.203^**^	0.169^**^	0.341^**^	0.319^**^	0.298^**^	0.493^**^	0.552^**^

Note. *p < 0.05. **p < 0.01

### Cross-lagged path analyses of social support, family resilience, and psychological resilience

**Fig. 3:**
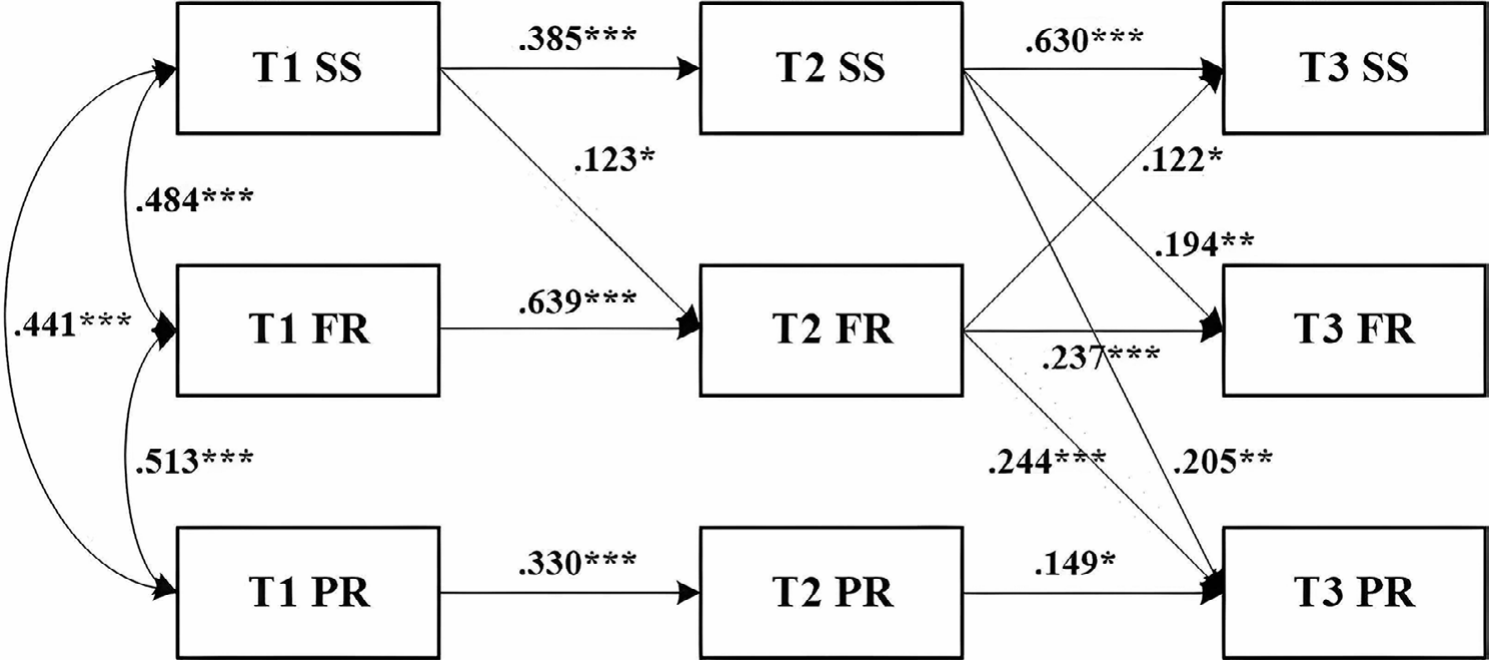
The cross-lagged modeling results and estimates (standardized) among social support, family resilience, and psychological resilience Note: SS indicates social support; FR,: family resilience; PR: psychological resilience; *p < 0.05,**p < 0.01,***p < 0.001. Employment status, education level, and duration of hemodialysis were controlled as covariates in the model

Given the variation in patient hemodialysis duration, we also controlled for it in cross-lagged analyses. Table 4 presents the fit indices and model comparison results of the five structural path models. The results showed that all models were well fitted. In conjunction with the chi-square difference test results indicate that model 5 is the optimal model [Δ*χ*^2^ (Δ*df*) = 10.441(4), *p*<0.05]. A total of five cross-lagged paths were significant at *P* < 0.05 in M5. All the insignificant cross-lagged paths were trimmed and resulted in the final model. As shown in Fig. 3, all autoregressive pathways of social support, family resilience, and psychological resilience were significant. That is, social support at T1 was significantly associated with T2 social support and T2 social support with T3 social support. The same pattern was shown for family resilience and psychological resilience. Autoregressive associations show measures of these three variables were stable along with time. Concerning the relationships between social support and family resilience, cross-lagged results showed that patients’ social support at T1 and T2 positively predicted family resilience at T2 [β = 0.123, 95% CI (0.026–0.244), *p* < 0.05] and T3 [β = 0.194, 95%CI (0.039–0.335), *p* < 0.01], respectively. What’s more, T2 family resilience predicted T3 social support. Finally, when it came to the relationships between psychological resilience, family resilience, and social support, the findings revealed that higher levels of social support [β = 0.205, 95%CI (0.049–0.354), *p* < 0.01] and family resilience [β = 0.244, 95%CI (0.119–0.359), *p* < 0.001] at T2 were associated with higher levels of psychological resilience at T3. This relationship was not bidirectional (i.e., psychological resilience exerted no effects on social support and family resilience).

**Table 4:** The goodness of fit statistics for the five models and model comparison

Model	χ^2^	df	χ^2^ /df	CFI	TLI	RMSEA	SRMR	Model comparison	Δχ^2^ (Δdf)	***P***
M1	63.799	25	2.552	0.957	0.888	0.079	0.081	-	-	-
M2	59.453	24	2.477	0.961	0.893	0.077	0.075	M2 vs. M1	4.35(1)	<0.05
M3	55.663	23	2.420	0.964	0.897	0.075	0.070	M3 vs. M2	3.79(1)	>0.05
M4	55.242	23	2.402	0.965	0.899	0.075	0.068	M4 vs. M2	4.211(1)	<0.05
M5	44.801	19	2.358	0.972	0.902	0.074	0.065	M5 vs. M4	10.441(4)	<0.05

## Discussion

### Stability and development of social support, family resilience, and psychological resilience in MHD patients

The findings indicated that MHD patients displayed some intertemporal stability in social support and psychological resilience. The possible reason is that the majority of the patients in our research had been on hemodialysis for a long time and had formed a solid psychological resilience and social support system [45]. Patients with MHD may not have undergone additional substantial traumatic experiences throughout this research’s brief duration, making it difficult to detect significant changes in psychological resilience and social support. Further study should extend the survey duration or focus on negative experiences [46–48] during the patient’s dialysis to gain deeper insights into the development of psychological resilience and social support. Notably, the psychological resilience of MHD patients in this study (57.35 ~ 59.55) at the three-time points was lower than that of frail older adults (68.5 ± 15.1) [49] and cancer patients (64.2 ~ 77.5) [50], indicating that the psychological resilience of MHD patients was consistently at a lower level. Therefore, healthcare professionals need to take effective measures to help patients improve their psychological resilience. Additionally, this study found that family resilience increased significantly at T3. Patients tend to underestimate the importance of their family advantage in the absence of outside distractions since the family is the major location of long-term survival and rehabilitation. The family resilience questionnaire’s positive statements about family let them appreciate the family’s beneficial power [51]. However, further studies should involve extended follow-up surveys or a combination of qualitative interviews to offer a deeper insight into the development of family resilience.

### The relationship between social support, family resilience, and psychological resilience in MHD patients

#### The relationship between social support and family resilience in MHD patients

By applying cross-lagged models, the present study examined the bidirectional relationship between social support, family resilience, and psychological resilience with three waves of data in MHD patients. Specifically, higher levels of social support significantly predicted better family resilience at T2 and T3. Moreover, family resilience at T2 positively predicted social support at T3. The results indicated that the impact of social support on family resilience is relatively consistent and stable over time. Social support [52–54] was recognized as an important resource that might affect families’ ability to endure and manage in the presence of a crisis. Likewise, Wong et al. [55] found that “drawing strength” was a major facilitator of family resilience in ICU patients. That is, ICU families gained emotional and informational support from their family members and other families of critically ill patients to break free from high emotional vulnerability and regain control, thereby increasing family resilience. However, family stress theory [56] states that the family’s perception of the stressful event and its available resources will determine how family stress and family capacity interact. This means that, in addition to social support, other factors such as the family’s cognitive evaluation of the crisis influence the dynamic process of family resilience. Future research could systematically explore the mechanisms by which factors such as family perceptions influence family resilience, given the impact of these aspects was not taken into account in this study.

Further, Walsh points out in the family resilience theory that family resilience can act as a buffer against risky crises and encompass dimensions such as socio-economic resources [18]. It means that more resilient families are more able to perceive existing social support from their surroundings (e.g. informational support from healthcare professionals, emotional support from extended family) and are able to mobilize united family members to expand their social support network to buffer against disease risk [33]. However, cross-lagged analyses revealed that T1 family resilience did not predict T2 social support. It is possible that the dynamics of family resilience may lead to different effect on social support, since the mechanisms influencing social support are complicated when MHD patients encounter multiple challenges and stresses in terms of treatment, finances, life, and psychology [57].

#### The relationship between social support and psychological resilience in MHD patients

Secondly, this study found that T2 social support predicted T3 psychological resilience significantly and positively, indicating that higher social support in MHD patients was associated with higher psychological resilience. This result confirms the main effect model of social support, which assumes that social support has a generally beneficial effect. In other words, regardless of whether an individual faces stressful situations or not, increasing social support is bound to significantly promote the development of the individual’s physical and emotional well-being. The resilience framework pointed out that favorable external resources can increase the level of psychological resilience [15]. Social support, as a critical available external resource, can help MHD patients develop the quality of resilience to cope with the complex disease process. A literature review [58] suggested that peer support was a potential resource that could provide additional emotional support and informational assistance to hemodialysis patients while enhancing their self-efficacy and self-management. Besides, social interactive support reduced the interpersonal burden of patients during hemodialysis and led to better clinical outcomes and psychosocial adjustment [59], aided by the fostering of resilience to reduce suffering.

#### The relationship between family resilience and psychological resilience in MHD patients

Regarding the relationships between family resilience and psychological resilience, the data showed that family resilience at T2 significantly predicted psychological resilience after T3. In contrast, psychological resilience in MHD patients did not predict family resilience at three months. The finding illustrated that family strength during a crisis situation affects the development of individuals within the family unit instead of individual development acting on family ability. This was consistent with previous cross-sectional [33] findings that higher family resilience enabled families to sufficiently leverage social resources and mobilize intrinsic personal resources, such as psychological resilience, to facilitate patients’ coping with stressful events positively. Conversely, lower family resilience contributed to exacerbating patients’ negative emotions and internal feelings of helplessness, exhibiting lower psychological resilience. Kukihara et al. [60] showed that family was one of the indispensable resources for patients. Strong family cohesion and support allow family members to assume their responsibilities and have a critical role in solving problems cooperatively and in helping patients to maintain a positive attitude toward life [61]. This provides evidence that hemodialysis patients recover from stress and further demonstrates that the ability of families to derive strength from traumatic experiences and cement emotional bonds can provide a supportive family environment, potentially increasing patients’ levels of psychological resilience.

However, the results showed that neither social support nor family resilience in T1 predicted psychological resilience in T2. The probable reason for this is that at the outset individual factors, such as symptom burden [41], psychological stress [8], and coping styles [62], have a greater impact on the patient’s psychological resilience. The social support and family resilience questionnaires in this study may have made patients aware of the important role of family and social factors. Due to the paucity of longitudinal studies on social support, family resilience, and psychological resilience in patients with MHD, future studies are needed to further validate the causal relationship between the aforementioned variables. At the same time, it is necessary to take into account the dynamic mechanisms of influence on psychological resilience of a variety of factors, such as disease factors and psychological burden.

### Relevance to clinical practice

The cross-lagged analysis of this study has important clinical implications for MHD patients’ intervention research, showing that greater social support and family resilience, along with time, are linked to an improvement in psychological resilience in patients with MHD. The finding reflects the importance of identifying and accessing social and family resources that may have a positive impact on an individual’s development. Specifically, healthcare professionals can develop family resilience-oriented interventions that raise the profile of family factors in psychological nursing interventions for hemodialysis patients. Tapping into the intrinsic strengths and power of families contributes importantly to remaining psychological resilience during and after MHD. Also, use social support preventatively and ongoing social support from the medical team or peers as a part of the patient’s standard of care throughout the dialysis process. Helping patients make better use of social resources is critical when families are learning to integrate the new and complex needs of patients with MHD into the family system. And extending these interventions to the patient’s family unit through psychosocial care providers may improve overall family resilience and thus enhance patient’s psychological resilience.

### Limitations

Several limitations of this study should be acknowledged. First, all data were based on patient self-reports, which may have individual subjective biases and potentially provide a more one-sided assessment of the three variables. Future research could use objective methods or collect data from multiple family members to get a more accurate picture of psychological resilience, family resilience, and social support, as well as more thoroughly assess the relationship between the three variables. Second, this study used convenience sampling and included a small sample size, with the sample limited to Wenzhou, Zhejiang Province, leaving deficiencies in representativeness. Thus, the generalizability to other populations with different sociodemographic backgrounds is unknown, and the findings of this study need to be viewed with caution. Third, due to time constraints, this study did not investigate the association between variables beginning at a specific exposure time (e.g., cognitive impairment, sleep disorders, social isolation), only three assessments were conducted. Future studies should consider using a long time or focusing on particular adversities to further understand the developmental trajectory of social support, family resilience, and psychological resilience in MHD patients and to show more complicated dynamic effects. Fourth, while this study controls for employment status and education level, there may be other confounding factors that were not accounted for, and caution is needed in interpreting the predictive relationships between the variables. Finally, this study only explored the relationship between family resilience, social support, and psychological resilience. Since previous research has found that psychological resilience is also influenced by other variables (such as self-efficacy, post-traumatic growth, and self-perceived burden [50, 63]), future studies should investigate the relationship between psychological resilience and these variables to form a more comprehensive picture of protective factors and risk factors for psychological resilience in MHD patients.

## Conclusions

In conclusion, this study found maintenance hemodialysis patients’ social support and psychological resilience remained relatively stable, whereas family resilience indicated a slight increasing trend. Social support and family resilience were mutually predictive, with clear and predictable consequences for subsequent psychological resilience in MHD patients. However, psychological resilience was not predictive of subsequent social support and family resilience. The current findings highlight the need for future studies to continue to assess the complex relationships between these variables using improved protocols (e.g., longer-term assessments, prospective qualitative studies). In addition, these results underscore the importance of improving patient resilience at the social and family levels. Clinical medical workers can build a system of social support networks and tailor family interventions to facilitate the construction of psychological resources for patients.

### Electronic supplementary material

Below is the link to the electronic supplementary material.

**Supplementary Material 1:** Baseline, forward causation, reversed causation, and reciprocal models for social support, family resilience, and psychological resilience, and STROBE checklist

## Data Availability

The datasets used and/or analyzed during the current study are available from the corresponding author on reasonable request.

## Abbreviations

ESRD: End-stage renal disease
MHD: Maintenance hemodialysis
STROBE: Strengthening the Reporting of Observational Studies in Epidemiology
MOS-SSS: Medical Outcomes Study-Social Support Survey
C-FRAS: Chinese version of the Family Resilience Assessment Scale
CD-RISC: Conner and Davidson resilience scale
SD: Standard deviation
*χ*^2^/*df*: Chi-square/degree of freedom
CFI: Comparative Fit Index
TLI: Tucker-Lewis Index
RMSEA: Root Mean Square Error of Approximation
SRMR: Standardized Root Mean Square Residual
