# Supplementary material for: Social support, family resilience and psychological resilience among maintenance hemodialysis patients: a longitudinal study
Source: BMC Psychiatry. 2024 Jan 26;24:76. doi: 10.1186/s12888-024-05526-4 (PMC10811847; doi:10.1186/s12888-024-05526-4)
Supplement: Supplementary file 1 — Supplementary Material 1: Baseline, forward causation, reversed causation, and reciprocal models for social support, family resilience, and psychological resilience, and STROBE checklist [file 12888_2024_5526_MOESM1_ESM.docx]

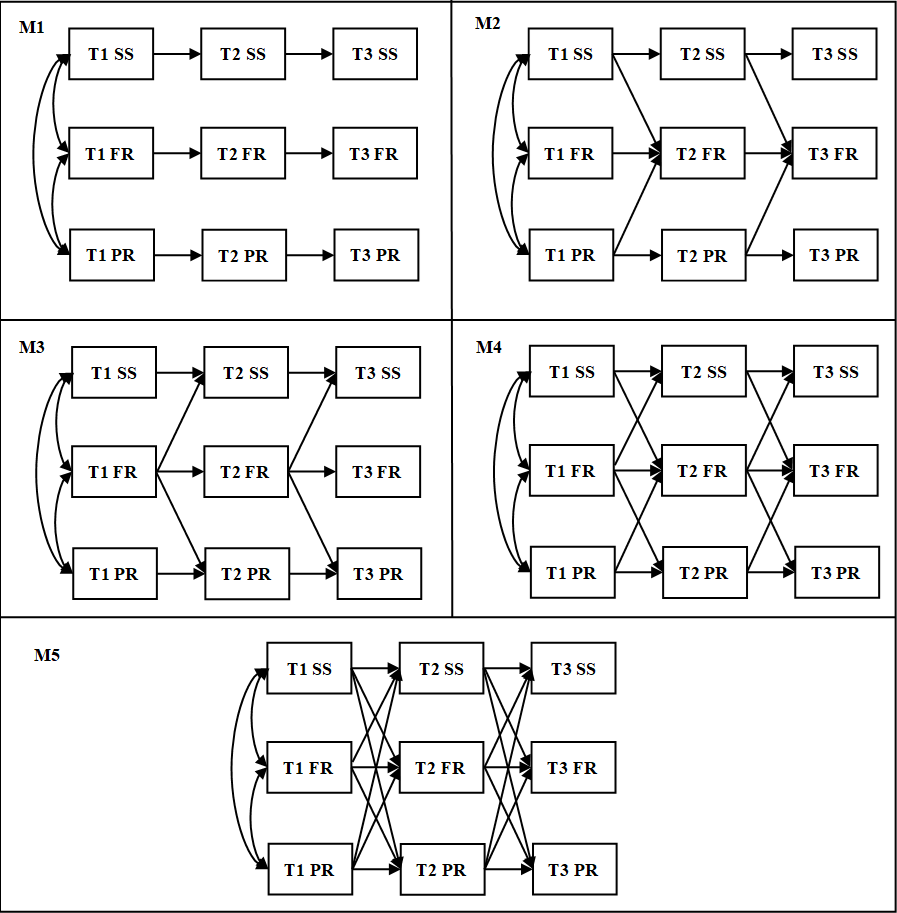


**Figure S1** Baseline, forward causation, reversed causation, and reciprocal models for social support, family resilience, and psychological resilience.

Note: SS indicates social support; FR, family resilience; PR, psychological resilience.

**Table S1** STROBE Statement—checklist of items that should be included in reports of observational studies

|  | Item No. | Recommendation | | Page  No. | | Relevant text from manuscript | |
| --- | --- | --- | --- | --- | --- | --- | --- |
| **Title and abstract** | 1 | (*a*) Indicate the study’s design with a commonly used term in the title or the abstract | | 1 | | “a longitudinal study” | |
|  |  | (*b*) Provide in the abstract an informative and balanced summary of what was done and what was found | | 1 | | “Methods:...” | |
| Introduction |  |  |  |  |  |  |  |
| Background/rationale | 2 | Explain the scientific background and rationale for the investigation being reported | | 2-3 | | “The prevalence of...” | |
| Objectives | 3 | State specific objectives, including any prespecified hypotheses | | 2-3 | | “this study hypothesizes that...”  “with the purpose of…” | |
| Methods |  |  |  |  |  |  |  |
| Study design | 4 | Present key elements of study design early in the paper | | 3 | | “This multi-center longitudinal...” | |
| Setting | 5 | Describe the setting, locations, and relevant dates, including periods of recruitment, exposure, follow-up, and data collection | | 3 | | “between September 2020 and July 2021 in hemodialysis centers in three comprehensive hospitals…” | |
| Participants | 6 | (*a*) *Cohort study*—Give the eligibility criteria, and the sources and methods of selection of participants. Describe methods of follow-up  *Cross-sectional study*—Give the eligibility criteria, and the sources and methods of selection of participants | | 3-4 | | “The eligibility criteria for participants...”  “…follow-up data in the hospital” | |
| Variables | 7 | Clearly define all outcomes, exposures, predictors, potential confounders, and effect modifiers. Give diagnostic criteria, if applicable | | 2-3 | | “Psychological resilience, defined as...” | |
| Data sources/ measurement | 8* | For each variable of interest, give sources of data and details of methods of assessment (measurement). Describe comparability of assessment methods if there is more than one group | | 4 | | “2.4 Measurements...” | |
| Bias | 9 | Describe any efforts to address potential sources of bias | | 4-5 | | “Based on previous studies [40, 41], patients’ gender…”  “…as covariates to control for potential confounding” | |
| Study size | 10 | Explain how the study size was arrived at | | 3 | | “Boomsma suggested a sample size of...” | |
| Quantitative variables | 11 | Explain how quantitative variables were handled in the analyses. If applicable, describe which groupings were chosen and why | | 4 | | “The statistical description...” | |
| Statistical methods | 12 | (*a*) Describe all statistical methods, including those used to control for confounding | | 4 | | “An independent sample t-test…” | |
|  |  | (*b*) Describe any methods used to examine subgroups and interactions | | NA | |  | |
|  |  | (*c*) Explain how missing data were addressed | | NA | |  | |
|  |  | (*d*) *Cohort study*—If applicable, explain how loss to follow-up was addressed  *Cross-sectional study*—If applicable, describe analytical methods taking account of sampling strategy | | NA | |  | |
|  |  | (*e*) Describe any sensitivity analyses | | NA | |  | |
| Results |  |  |  |  |  |  |  |
| Participants | 13* | (a) Report numbers of individuals at each stage of study—eg numbers potentially eligible, examined for eligibility, confirmed eligible, included in the study, completing follow-up, and analysed | 3 | | “Twenty-two of the 280...” | |  |
|  |  | (b) Give reasons for non-participation at each stage | Figure 1 | |  | |  |
|  |  | (c) Consider use of a flow diagram | Figure 1 | |  | |  |
| Descriptive data | 14* | (a) Give characteristics of study participants (eg demographic, clinical, social) and information on exposures and potential confounders | 5  Table 1 | | “The MHD patients' ages ranged…” | |  |
|  |  | (b) Indicate number of participants with missing data for each variable of interest | NA | |  | |  |
|  |  | (c) *Cohort study*—Summarise follow-up time (eg, average and total amount) | 3 | | “... hree months (T2) and six months (T3)” | |  |
| Outcome data | 15* | *Cohort study*—Report numbers of outcome events or summary measures over time | Figure 1 | |  | |  |
|  |  | *Cross-sectional study—*Report numbers of outcome events or summary measures | NA | |  | |  |
| Main results | 16 | (*a*) Give unadjusted estimates and, if applicable, confounder-adjusted estimates and their precision (eg, 95% confidence interval). Make clear which confounders were adjusted for and why they were included | 5 | | “…employment status and education level as covariates”  “Given the variation in patient hemodialysis duration, we also controlled” | |  |
|  |  | (*b*) Report category boundaries when continuous variables were categorized | NA | |  | |  |
|  |  | (*c*) If relevant, consider translating estimates of relative risk into absolute risk for a meaningful time period | NA | |  | |  |
| Other analyses | 17 | Report other analyses done—eg analyses of subgroups and interactions, and sensitivity analyses | | NA | |  | |
| Discussion |  |  |  |  |  |  |  |
| Key results | 18 | Summarise key results with reference to study objectives | | 7-9 | | “The findings indicated...” | |
| Limitations | 19 | Discuss limitations of the study, taking into account sources of potential bias or imprecision. Discuss both direction and magnitude of any potential bias | | 10 | | “Several limitations of this study should be…” | |
| Interpretation | 20 | Give a cautious overall interpretation of results considering objectives, limitations, multiplicity of analyses, results from similar studies, and other relevant evidence | | 7-9 | | “The probable reason for this is…” | |
| Generalisability | 21 | Discuss the generalisability (external validity) of the study results | | 10 | | “…has important clinical implications...” | |
| Other information |  |  |  |  |  |  |  |
| Funding | 22 | Give the source of funding and the role of the funders for the present study and, if applicable, for the original study on which the present article is based | | 11 | | “The work was supported by...” | |

STROBE Statement—checklist of items that should be included in reports of observational studies

*Give information separately for cases and controls in case-control studies and, if applicable, for exposed and unexposed groups in cohort and cross-sectional studies.

**Note:** An Explanation and Elaboration article discusses each checklist item and gives methodological background and published examples of transparent reporting. The STROBE checklist is best used in conjunction with this article (freely available on the Web sites of PLoS Medicine at http://www.plosmedicine.org/, Annals of Internal Medicine at http://www.annals.org/, and Epidemiology at http://www.epidem.com/). Information on the STROBE Initiative is available at www.strobe-statement.org.
